# The enriched mind: cognitive stimulation and behavior in non-human primates

**DOI:** 10.3389/fvets.2025.1724923

**Published:** 2025-12-12

**Authors:** Daniel Mota-Rojas, Alexandra L. Whittaker, Genaro A. Coria-Avila, Adriana Domínguez-Oliva, Cécile Bienboire-Frosini, Ismael Hernández-Avalos, Cuauhtémoc Chávez, Adriana Olmos-Hernández, Julio Martínez-Burnes, Ricardo García-Herrera, Patricia Mora-Medina, Temple Grandin

**Affiliations:** 1 Neurophysiology, Behavior and Animal Welfare Assessment, DPAA, Universidad Autónoma Metropolitana (UAM), Mexico, Mexico; 2 School of Animal and Veterinary Sciences, University of Adelaide, Roseworthy, SA, Australia; 3 Instituto de Investigaciones Cerebrales, Universidad Veracruzana, Xalapa, Mexico; 4 EPLFPA-Avignon, Avignon, France; 5 Facultad de Estudios Superiores Cuautitlán, FESC, Universidad Nacional Autónoma de México (UNAM), Cuautitlán, State of Mexico, Mexico; 6 Departamento de Ciencias Ambientales, CBS Universidad Autónoma Metropolitana-Lerma, Lerma de Villada, Estado de México, Mexico; 7 Division of Biotechnology-Bioterio and Experimental Surgery, Instituto Nacional de Rehabilitación Luis Guillermo Ibarra Ibarra (INR-LGII), Mexico, Mexico; 8 Instituto de Ecología Aplicada, Facultad de Medicina Veterinaria y Zootecnia, Universidad Autónoma de Tamaulipas, Ciudad Victoria, Mexico; 9 División Académica de Ciencias Agropecuarias, Universidad Juárez Autónoma de Tabasco, Villahermosa, Mexico; 10 Department of Animal Science, Colorado State University, Fort Collins, CO, United States

**Keywords:** environmental enrichment, cognitive skills, great apes, monkeys, animal welfare

## Abstract

Non-human primates (NHPs) possess high cognitive abilities that enable them to respond effectively to complex social, ecological, and psychological challenges. These abilities need to be stimulated in non-human primates under human care in zoos, conservation centers/sanctuaries, or research facilities, where stereotypies and behavioral and/or psychobiological disturbances are frequently associated with captivity. Cognitive enrichment (CE), a type of environmental enrichment that facilitates decision-making skills, problem-solving, and control over the environment, is one way to promote primate welfare by encouraging engagement in cognitive tasks. Currently, non-technological and technological cognitive enrichments are implemented for NHPs under human care to enhance positive behavioral responses and prevent negative emotional states such as boredom or stress. The present review aims to analyze the benefits that CE provides to NHPs (including monkeys and great apes), highlighting its relevance to research, conservation, and ethical management in controlled environments.

## 1 Introduction

Environmental enrichment (EE) has been defined as “an animal husbandry principle that seeks to enhance the quality of captive animal care by identifying and providing the environmental stimuli necessary for optimal psychological and physiological wellbeing” (1). EE is a facilitator of animal welfare that provides physical, sensory, social, and cognitive stimuli to animals, promoting species-typical behaviors, especially in species with highly complex cerebral processing, such as non-human primates (NHPs) (2). NHPs possess very complex behavioral repertoires and a high level of brain plasticity, which requires an enriched and adequate environment for their proper expression (3–5). In particular, cognitive enrichment (CE) elicits functions such as associative learning, problem-solving (visual navigation and planning), metacognition, perceptual illusions, and numerical and quantitative judgments (6). As described by Huskisson et al. (7), providing a cognitively enriched environment aims to mimic the ecological and social challenges animals would face in their natural habitats. This is relevant for NHPs under human care in zoos, conservation centers/sanctuaries, or research facilities, where stereotypies and behavioral and/or psychobiological disturbances are frequently associated with captivity (8, 9).

Under the “Five Domains” model, large-brained mammals require mentally stimulating environments to avoid negative emotional states such as frustration, anxiety, or depression (10–12). CE has widely been demonstrated to promote behavioral and neurophysiological homeostasis (2), particularly in NHPs (5, 13). Several studies performed in chimpanzees (*Pan troglodytes*), rhesus monkeys (*Macaca mulatta*), Tufted capuchins (*Sapajus apella*), and common marmosets (*Callithrix jacchus*) highlight their ability to perform complex cognitive operations, such as spatiotemporal working memory measured by delayed non-match to sample, sensorimotor tasks (14), stimulus-reward association learning measured by object discrimination tests (15), and emotional contagion (16–18). These studies demonstrate these animals' advanced cognitive abilities and the need for constant mental stimulation to preserve their emotional wellbeing (5, 8, 11, 19).

The effect that CE has on NHPs can be directly observed on brain development and function (20). Different studies have demonstrated that CE induces significant changes in brain anatomy and physiology. Examples include effects on aspects of neuroplasticity such as changes in dendritic spine growth, cortical thickness, dendritic branching, and presynaptic vesicle number (21). CE also promotes hippocampal neurogenesis and synaptogenesis, improving synaptic efficiency through the synaptophysin expression, and upgrading neuronal circuits associated with attention, working memory, and decision making in the anterior cingulate cortex, dorsolateral prefrontal cortex, and pulvinar thalamic nucleus (22). Continuous exposure to mental challenges, even in adulthood, delays cognitive decline by reducing neuronal apoptosis to prevent the development of disorders such as senile dementia (23), modulates the main stress-related systems (hypothalamic-pituitary-adrenal and sympathetic adrenomedullary axes), and increases behavioral resilience (24).

Currently, CE programs for NHPs include the application of problem-solving activities as novel strategies (8, 9, 25). The introduction of experimental paradigms such as touchscreen interfaces, video demonstrations (26), eye-tracking devices (27), behavioral tasks, puzzles with rewards (14), or tactile or 3D digital platforms (28) has proven to be highly effective in stimulating curiosity in primates through the activation of the mesolimbic dopaminergic system. Technological devices provide broad benefits through providing the ability to monitor latency and duration of behavioral responses, as well as assessment of problem-solving efficiency and cognitive flexibility (29).

Implementing CE programs inside facilities housing NHPs is an ethical obligation aimed at improving the welfare of captive animals (7) since depriving animals of opportunities to meet their biological needs might be considered a form of negligence (29). Furthermore, by integrating CE into primates' daily routines, stress levels are minimized, voluntary participation increases, and the individual's biological needs are met. All this can be achieved in a noninvasive manner (29), and for animals used in research, does not come at the expense of the reproducibility and validity of the research (5, 8, 9, 19). This review aims to summarize the benefits of CE for NHPs (monkeys and great apes), highlighting its relevance to research, conservation, and ethical management in controlled environments.

## 2 Why do non-human primates require cognitive enrichment?

The answer lies in the fact that many taxa possess an ancestral neural system that has evolved to direct behavior toward stimuli with incentive value, enabling them to seek resources that enhance biological fitness through the capacity to predict beneficial outcomes based on their desires. This so-called SEEKING system, a primary emotional circuit described by Panksepp (30), motivates exploration and reward acquisition, directly supporting survival and reproductive success.

In the wild, this system is continuously engaged through natural challenges such as foraging for varied food sources, navigating complex environments, avoiding predators, and participating in social and tactical interactions. Its activation is not merely beneficial but essential: it transforms passive existence into an active, adaptive process, enabling animals to learn about their surroundings, form predictive associations, and experience positive affective states that are fundamental to wellbeing (31).

In enclosed environments, however, opportunities for natural stimulation are often drastically reduced. The lack of unpredictability, cognitive challenge, and opportunities for exploratory control can lead to under-stimulation of the SEEKING system, resulting in apathy, boredom, or conversely, aberrant compulsive behaviors when the system becomes dysregulated (32). Neurobiologically, the SEEKING system is primarily supported by dopaminergic mesolimbic and mesocortical pathways, although many other neurotransmitters may be involved. Evidence indicates that this system responds with phasic (rapid) dopamine release within milliseconds, signaling reward prediction errors (33), while tonic (slow) dopamine modulates sustained motivation and attention (34).

Thus, this system drives desire, facilitates associative learning, and enhances cognitive engagement through mechanisms of incentive salience in all mammals. NHPs have an expanded prefrontal cortex, which supports advanced executive functions such as future planning, abstract reasoning, and behavioral inhibition. This neural enhancement likely enables NHPs to foresee longer-term future outcomes based on more distant past experiences, allowing them to engage in behaviors such as tool use, tactical deception, and multi-step problem solving. The increased cortical integration also heightens the need for cognitive stimulation, making environmental enrichment not only beneficial but also critical for psychological wellbeing. Therefore, understanding the neuroethological basis of the SEEKING system is paramount for designing evidence-based CE that supports not only natural behavior but also positive emotional states and functional neurological health in captive primates.

Effective CE strategies are designed explicitly to activate this circuit. They introduce elements of uncertainty, problem-solving, and contingent reward that mimic natural seeking behaviors, thereby promoting dopamine-mediated states of curiosity and anticipatory excitement. Conversely, environments that are overly predictable or lack opportunities for agentic control fail to adequately stimulate this system, resulting reduced dopaminergic tone and behavioral signs of poor welfare. Accordingly, abnormal hyperactivity of the SEEKING system, often observed in sterile or frustrating environments, can also manifest as stereotypic or compulsive behaviors, evidence of a system struggling to adapt to suboptimal conditions (35).

## 3 Definition of cognitive enrichment

The term CE refers to a subset of EE in which the provision of objects, materials, and/or sensory stimulation enhances the environment and promotes cognitive functioning in animals (8, 36). It involves cognitive skills that are developed by providing the opportunity to solve problems and control elements of the environment (37). Most enrichment programs use rewards that can be intrinsic (such as the feeling of satisfaction when solving a task) or extrinsic (such as food) (38). The reward type must be appropriate for the species; however, most enrichment programs with captive animals use food rewards (39). Moreover, for NHPs under human care in zoos, primate service centers, and research institutions, novelty, controllability, and difficulty of the challenge are crucial (40). Non-challenging environments have been proposed as the main underlying factor of boredom and, consequently, self-directed behaviors or stereotypies (41).

In order to develop appropriate CE, a strong knowledge of the cognitive abilities of the taxa is needed (42). For NHPs, two main types of CE are reported (39). CE_skill_, primarily studied in laboratory settings, aims to slow down the rate of cognitive decline or enhance the cognitive abilities of primates (2). An example of this is the study by Kim et al. (43), where Rhesus macaques (*Macaca mulatta*) were subjected to a finger maze test. During the enrichment, monkeys received a reward for selecting the correct route. This learning test was performed 2 days, 10 times a day, and 2 months later, a memory test was conducted by repeating the same procedure. The results showed that the average success rate on the learning test was 89.6%, while in the memory test it was 84.6%. This type of CE provides information regarding the cognitive skills of animals as well as their application in evaluating the cognitive function of learning and memory in NHPs.

On the other hand, CE_welfare_ is the most common type of CE. Its objective is to improve animal welfare, focusing on both immediate emotions and long-term effects through physiological indicators of stress (2). Hirskyj-Douglas and Kankaanpää (44) evaluated the performance of zoo white-faced sakis (*Pithecia pithecia*) aged 4–22 years old when exposed to a computer-enabled screen system with visual elements. The device consisted of a tunnel-shaped structure with a screen, a camera, and proxemic detectors embedded within the wall. When the device detected a saki, the system showed a video on the screen inside the tunnel. When assessing sakis' behavioral response to the CE, the authors found a significant decrease in scratching (by 59.91%). The application of this type of CE suggests that welfare improved because scratching is a stress-related displacement behavior in monkeys (45). These studies demonstrate that implementing environmental changes is often accompanied by a corresponding behavioral response. Therefore, in many cases, the effectiveness of CE is reflected in a decrease in atypical or stereotypical behaviors in NHPs, as discussed below (46). Table 1 shows some examples of CE according to the previously discussed categories.

**Table 1:** Examples of the two types of CE in NHPs.

**Type**	**Aim**	**Devices**	**Primary outcomes**	**Examples**
CE_skill_	Maintain specific cognitive abilities, often for long-term benefits like slowing cognitive decline or teaching survival skills	• Finger maze	Learning success	(43)
		• Puzzle feeder • Food puzzles • Ant-fishing simulation	Problem-solving skills and encouragement for foraging	(48, 53, 59, 62, 65, 73, 91)
CE_welfare_	Improve the animal's current state and welfare	• Digital screens • Videotapes • Virtual painting • Virtual forest	Playful interactions with the devices	(44, 85, 87, 88)
		• Foraging/grooming boards or boxes	Encourages foraging	(49, 50, 66)

## 4 Cognitive enrichment in monkeys

**Figure 1:**
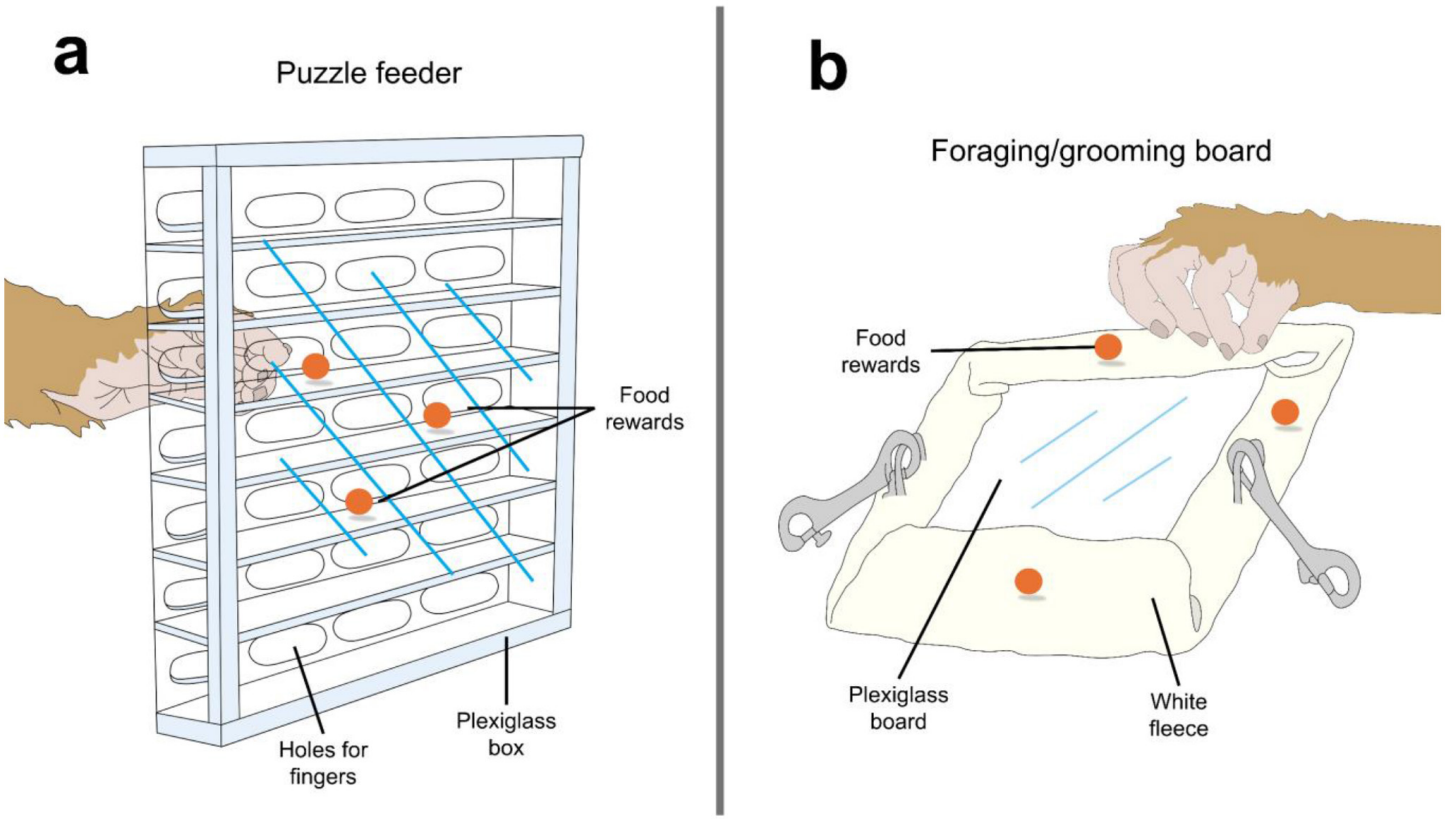
Schematization of cognitive enrichments used for monkeys under human care. **(a)** Puzzle feeders promote foraging behaviors and engage animals in cognitive activities. **(b)** Foraging/grooming boards are an option for macaques housed in research centers. Boards can also have divots and holes to encourage foraging. Original images made with information retrieved from Bayne et al. (49) and Lee et al. (48).

Primates are commonly used in biomedical research, especially in safety testing, yet their use presents key ethical challenges. Housing NHPs in laboratories should allow them to satisfy their behavioral needs while considering the risks of confounding experimental protocols. Given their high level of cognition and propensity to develop atypical behaviors in captivity, CE has become established as a method to reduce these behaviors. The effects may even be so great as to positively affect animals whilst singly-housed, a condition that represents a significant impediment to welfare, as shown by Schapiro et al. (47), who found that foraging behaviors increased through puzzle and mat provision. Figure 1 schematizes some of the enrichment devices used for monkeys and discussed in the present section (48, 49).

A key goal of CE is to encourage species-typical behavior (46). This was studied by Gronqvist et al. (50) in Javan gibbons (*H. moloch*) housed in an animal park and exposed to three different enrichment devices over 8 weeks. The devices included scent mats, foraging boxes, and boomer balls of different colors (red and blue). The scented mats were soaked in one of five scents (peppermint, almond, cinnamon, ginger, and mixed Allspice powder) and were changed daily. Foraging boxes contained food inside, and animals could retrieve it by hand. The authors found that, when compared with non-enriched animals, the three types of CE significantly increased foraging behavior by up to 4.8%. Additionally, the results showed that gibbons spent a larger amount of time interacting with the foraging box (0.082 ± 0.031 times/min) compared to the boomer balls and scent mats (0.026 ± 0.011 and 0.015 ± 0.009 times/min, respectively). This showed a predilection for the foraging device, as it was cognitively challenging and associated with appetitive and consummatory behaviors. This is relevant for the species as gibbons, in the wild, spend 70% of their day foraging (51).

CE also aims to reduce the expression of atypical behaviors or stereotypies, as shown in the Ogura et al. (52) study, which projected YouTube videos onto a laptop to laboratory Japanese macaques (*Macaca fuscata*). The videos combined media involving “Japanese macaques,” “people,” and “animation,” and were played for 30 days. The results showed that when individually housed monkeys did not interact with the monitor; the frequency of atypical behaviors (autoerotic stimulation, self-biting, hair plucking, rubbing/licking bars, and licking hands) was ~22. In contrast, during the video reproduction, the frequency of atypical behavior decreased to 12. Similarly, in female laboratory rhesus monkeys, Lee et al. (48) evaluated the short-term effect of implementing a puzzle feeder on reducing stereotypic behaviors, such as hand-rubbing the cage door by hand, pacing, and bouncing. The puzzle feeder had several holes placed in front of the box, along with horizontal and vertical Plexiglas pieces that could be arranged to form different mazes from which monkeys could retrieve the food with their fingers (Figure 1a). After 16 weeks, stereotypies were almost eliminated. Additionally, Novak et al. (53) reported that implementing puzzle feeders in individually housed monkeys reduced pacing and rocking but did not affect self-injurious behavior such as self-biting.

A significant reduction in the presentation of atypical behaviors was also reported by Bayne et al. (49) in individually housed female and male rhesus monkeys when exposed to foraging/grooming boards. The board consisted of a Plexiglass board covered with fleece, on which food particles of different flavors were rubbed to promote foraging (banana, cherry, strawberry, and orange) (Figure 1b) (49). After 6 months, the authors found that the duration of atypical behaviors (e.g., circling, pacing, self-directed, and cage-directed) decreased significantly (from 316 to 36 s) during the interaction with the enrichment. Likewise, the frequency of atypical behaviors also reduced from 16.4 to 11.0. From a mechanistic perspective, Lutz et al. (54) suggest that atypical behaviors such as self-injurious behaviors are related to increases in cortisol and other hypothalamic-adrenal responses, reflecting stress. Thus, CE might serve as a technique to reduce cortisol and the stress response. This idea is supported by Xinbo et al. (55), who showed that cortisol in snub-nosed monkeys (*Rhinopithecus roxellana*) declined following enrichment provision (to ~2.5 ng/mg).

Whilst CE has been proposed as a method to promote good welfare, there may be some negative sequelae that need to be considered. A major challenge occurs when providing enrichment to social groups of primates, since the devices may generate competition over the resource. For example, Jacobson et al. (56) showed that in zoo-housed Japanese macaques (*Macaca fuscata*), there was increased aggression on days when CE was provided, albeit that rate of aggression was still a very low level (< 0.02 events/min). Likewise, mirror enrichment has been suggested as a social and sensory enrichment for captive primates, such as research monkeys. The self-image in the reflection is often perceived as a conspecific, which might elicit positive affiliative responses. However, as de Groot and Cheyne (57) note, aggression is frequently observed, and a decrease in atypical behaviors are rarely recorded, requiring individual assessment of effect of the enrichment on welfare.

Considering that primate species are extraordinarily variable, it is estimated that their responses to environmental changes are also variable. Therefore, for optimal enrichment strategies in monkeys, it is essential to understand the species' natural history, as techniques that work for one species may not be beneficial for others (Table 2) (46).

**Table 2:** Summary of discussed studies addressing CE for monkeys.

**Species**	**Setting**	**Device**	**Sample size**	**Duration**	**Outcomes**	**References**
Rhesus monkeys (*Macaca mulatta*)	Lab	Foraging/grooming boards	8	6 months	↓ Atypical behavior	Bayne et al. (49)
Javan gibbons (*Hylobates moloch*)	Zoo	Scent mats Foraging boxes Boomer balls	10	8 weeks	↑ Foraging	Gronqvist et al. (50)
Japanese macaques (*Macaca fuscata*)	Zoo	Touchscreen	12	15 months	↑ Aggression	Jacobson et al. (56)
Rhesus monkeys (*Macaca mulatta*)	Lab	Puzzle feeder	5	16 weeks	↓ Stereotypic behavior	Lee et al. (48)
Rhesus monkeys (*Macaca mulatta*)	Lab	Puzzle feeder	15	6 weeks	↓ Pacing ↓ Rocking	Novak et al. (53)
Japanese macaques (*Macaca fuscata*)	Lab	Youtube videos	10	30 days	↓ Atypical behavior	Ogura et al. (52)
Snub-nosed monkeys (*Rhinopithecus roxellana*)	Zoo	Trees to climb	7	4 months	↓ Idle time ↓ Cortisol	Xinbo et al. (55)

↑, significant increase; ↓, significant decrease; N/S, not specified.

## 5 Cognitive enrichment in great apes

Nonhuman great apes, including chimpanzees (*Pan troglodytes*), gorillas (*Gorilla gorilla*), orangutans (*Pongo pygmaeus*), and bonobos (*Pan paniscus*), are characterized by their notorious capacity to explore their environment, their intelligence, and cognitive abilities (37, 58). In the wild, great apes are exposed to cognitively stimulating environments and to complex social, ecological, and psychological challenges that require decision-making skills (36, 59). Furthermore, great apes explore novel objects by using and creating tools due to their high manipulative skills, which are directly related to their inherent problem-solving abilities and their close genetic resemblance to humans (36, 37). For example, Keller and DeLong (60) mention that orangutans have the same ability as 5–to 10-year-old children in using stick tools to solve mazes in a puzzle box.

**Figure 2:**
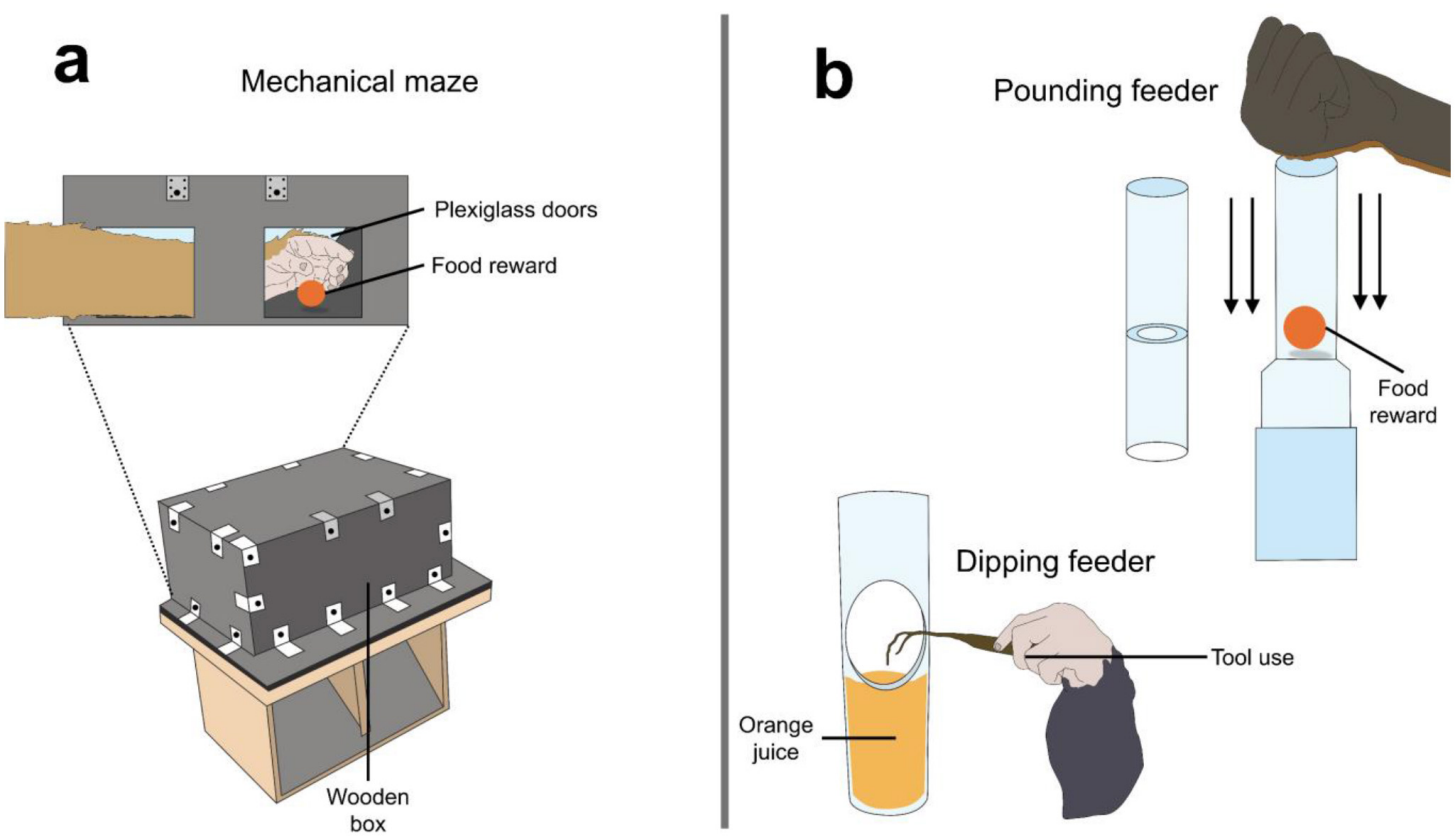
Schematization of cognitive enrichments for great apes. **(a)** A mechanical maze where apes had to understand how to obtain the reward indirectly. **(b)** Pounding and dipping feeders promote natural behaviors such as ant-fishing and tool use. Original images made with information retrieved from Vlamings et al. (64) and Yamanashi et al. (69).

Great apes under human care frequently show atypical and pathological behaviors (e.g., self-harm or repetitive behaviors) when experiencing boredom, anxiety, and stress under impoverished husbandry conditions, non-stimulating environments, or due to the lack of control over their environment (37, 59, 61). Thus, it is essential to create environments that mimic the daily challenges faced by great apes in the wild, thereby eliciting species-typical behaviors (62, 63). For example, Vlaming et al. (64) mention that great apes can solve mechanical and computerized mazes. These mazes consisted of wooden boxes with transparent Plexiglass doors at the front (Figure 2a) (64). Animals could insert their entire arm to reach the food reward (a banana). Using these devices, the authors found that apes solved the mazes by indirectly reaching the food through the empty door, thereby avoiding the food reward from falling. CE in great apes has been shown to improve the physical and psychological wellbeing of animals by engaging them in problem-solving activities, minimizing stress, reducing the expression of atypical, agonistic, and pathological behaviors, and increasing the expression of voluntary natural behaviors (36, 59, 61, 62, 65).

**Figure 3:**
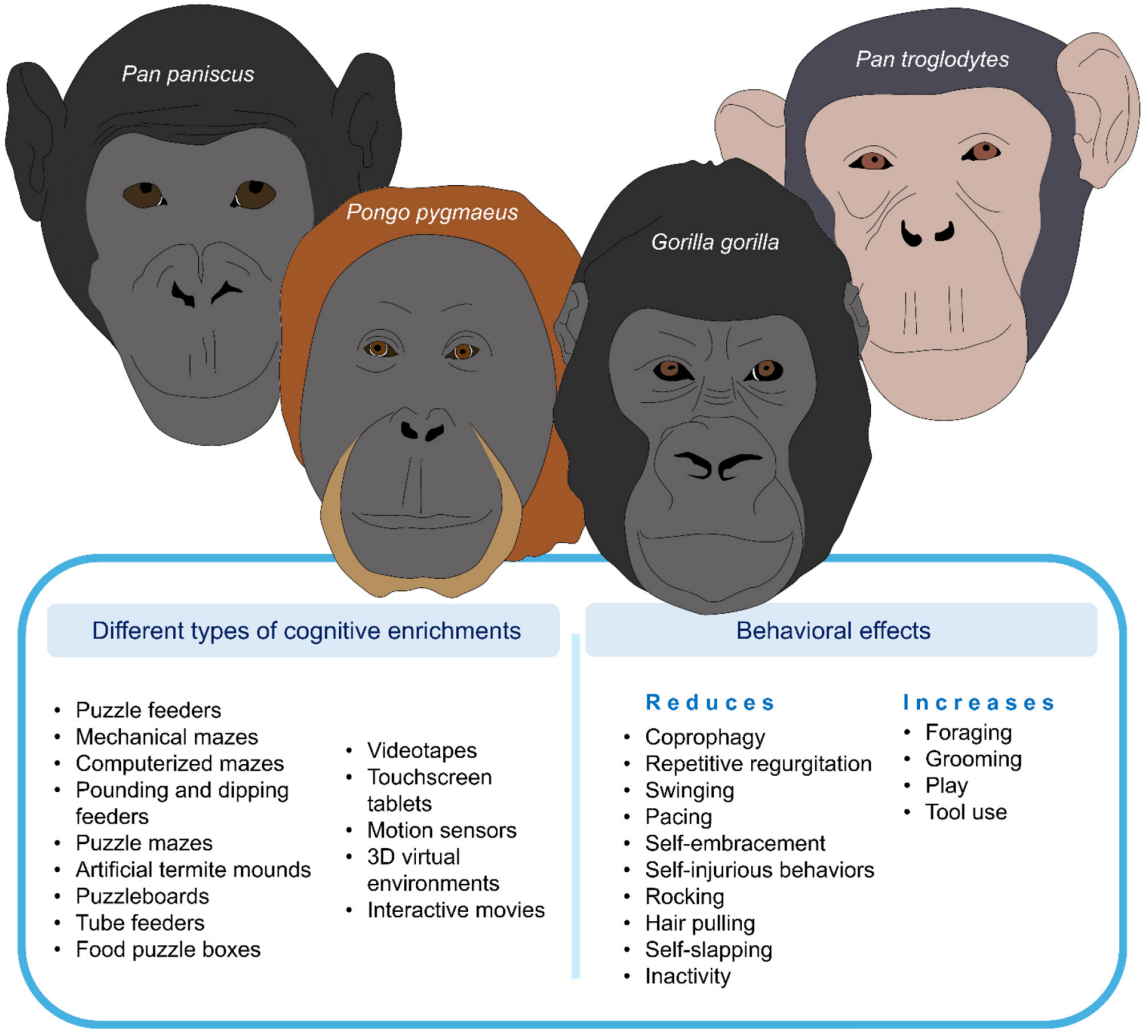
Cognitive enrichment in great apes: types of stimuli and associated behavioral modifications.

As observed in monkeys, CE for great apes includes food-based rewards to promote foraging, as great apes spend ~60% of their daily activity budget foraging (66, 67). Foraging and feeding involve high levels of tool use, locomotion, cognition, and dexterity (37, 68). Therefore, devices or puzzles that provide appropriate food/foraging opportunities have been reported to have several benefits, such as increasing positive emotion-related responses (e.g., grooming, play, tool use) and decreasing atypical responses (coprophagy, repetitive regurgitation, swinging, and self-embracement; Figure 3) (59, 62).

Chimpanzees are the most studied great apes under human care (67), and several CE devices have been developed for them, particularly those that promote tool use and food reward, to reduce the presentation of stereotypies. For example, chimpanzees housed in indoor/outdoor enclosures at a science park were exposed to foraging enrichments to evaluate their behavioral responses during 270 h of observation (65). The enrichment consisted of adding foods that required more processing time for consumption (e.g., corn on the cob), a dispenser with monkey biscuits, and food spread in the enclosure. Monkeys were also exposed to three food puzzle devices filled with popcorn, sunflower seeds, and peanuts. The devices required manipulation to obtain food items. It was found that the percentage of agonistic (e.g., bared-teeth screams, bites, tug/hit, among others) and atypical behaviors (coprophagy, rocking, hair pulling, self-slapping, among others) significantly reduced by 48% and 63.6%, respectively, from baseline values to when animals received enrichment. Feeding and social grooming increased by 54.3 and 31.7%, respectively. Similarly, Yamanashi et al. (69) tested two PVC pipe feeders in zoo-housed male and female chimpanzees, where tools were required to obtain food rewards in pounding and dipping feeders, aiming to stimulate both pestle-pounding and ant-dipping behaviors during two sets of 2 months each (Figure 2b). Dipping feeders were filled with fruit juice diluted with water. The juice was accessible by soaking and licking twigs. In the pounding feeder, peanuts, cabbage, bananas, and steamed sweet potatoes were accessible after chimpanzees pushed the food to the bottom of the feeder. Behavioral evaluations in adult individuals revealed that the frequency of self-directed behaviors (e.g., self-slapping) decreased significantly from ~0.1 to 0.03. A similar response was observed for atypical behaviors (regurgitation, feces smearing), decreasing from 0.02 to 0. The frequency of inactive time also significantly decreased from 0.6 to 0.55. Pestle-pounding and ant-dipping are behaviors that wild chimpanzees learn from a young age, particularly in Bossou, Guinea. Their objective is to consume the apical buds of oil palm trees and army ants (70, 71). Thus, exposing chimpanzees to EE motivates natural behaviors that benefit their mental state.

**Figure 4:**
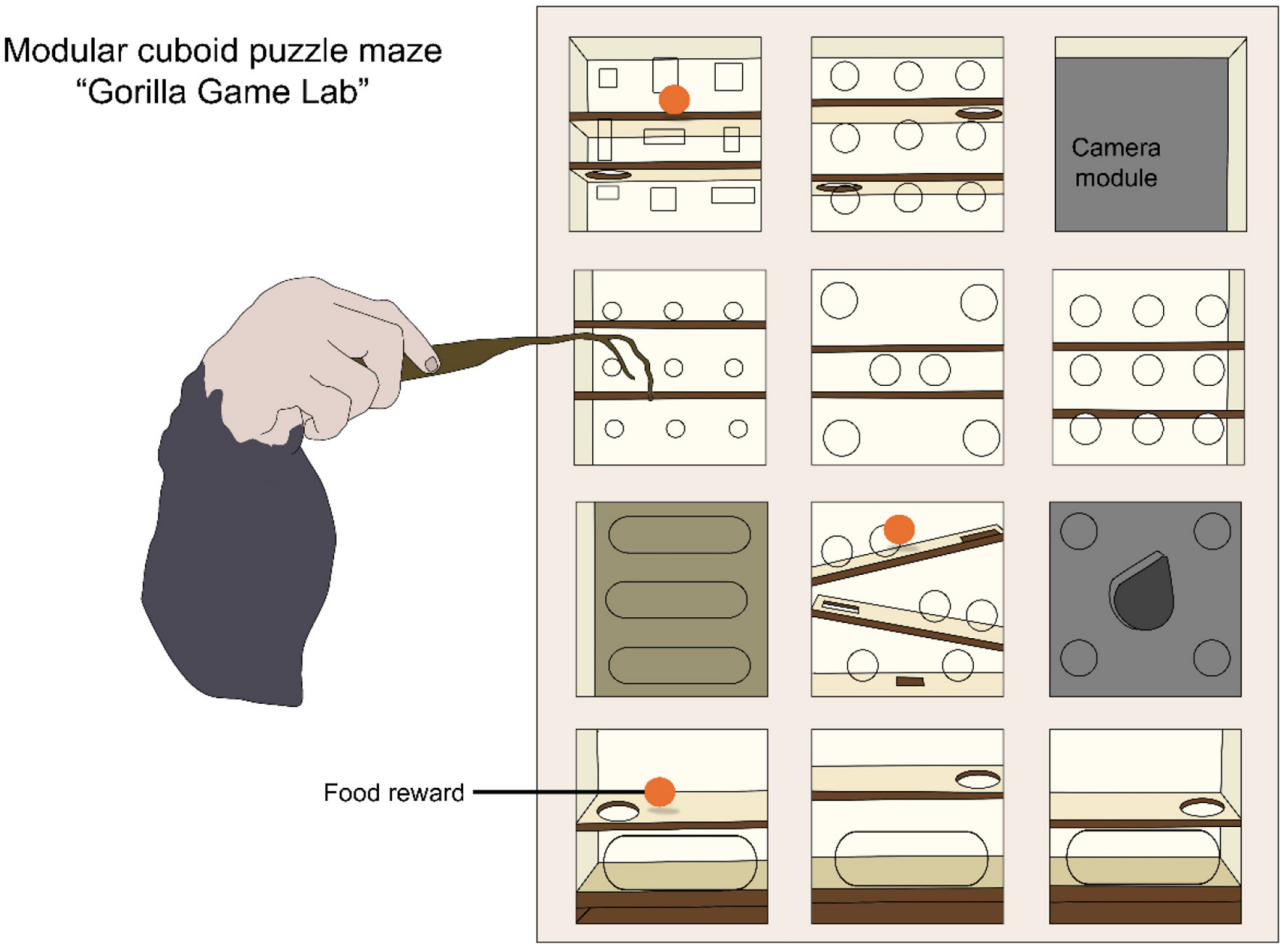
Modular cuboid puzzle maze as a cognitive enrichment tool for gorillas under human care. Puzzles where animals need to move the food reward through a maze serve as a method to increase foraging behavior and solving skills. Original image made with information retrieved from Clark et al. (73).

Gray et al. (72) and Clark et al. (73) developed a modular cuboid puzzle maze (called “Gorilla Game Lab”) to enrich the enclosures of Western lowland gorillas. Animals could move food rewards (unshelled peanuts) from the top row to the bottom, with their fingers or tools, through twelve different modules with ramps or small shelves as obstacles (Figure 4) (73). The authors evaluated behavioral responses and reported that gorillas interacted with the device for up to 2 h, without a decline in use. No aggressive or atypical/aberrant behavior (e.g., rock, regurgitate, pluck hair, or self-injurious behaviors) was observed during device use. In 64.2% of cases, the gorillas used tools to obtain the reward. Interestingly, the authors found that, after exposure to the CE, younger gorillas played significantly more than older subjects, which might help to design the EE based on individual age.

Zaragoza et al. (59) also implemented several enrichment methods for 6 weeks to promote foraging and termite fishing behavior in male and female chimpanzees and gorillas. Items included cardboard boxes filled with newspapers and food (e.g., dried grapefruits, fruit, and vegetables), and artificial termite mounds made with PVC tubes containing honey and dried grapefruits, rubber balls, mirrors, nautical hollow buoys with dried fruits, and fire hoses. According to the behavioral evaluation, inactivity time significantly decreased in chimpanzees and gorillas when exposed to enrichment (~30% in both species), as well as the frequency of atypical behaviors (coprophagy, repetitive regurgitation, swinging, and self-embracement; from 8 to below 5%). In contrast, in both species, feeding, social, locomotive, and exploratory behaviors increased significantly in enriched groups (by up to 35%). When comparing fecal cortisol concentrations, gorillas recorded significant increases from 20 to ~35 ng/mg during the enrichment phase (59). Increases in salivary cortisol concentrations were also recorded by Behringer et al. (74) in groups of bonobos, western lowland gorillas, and Sumatran orangutans (*P. abelii*; up to 6.6 ng/ml) when exposed to EE compared to routine days. Although elevated cortisol concentrations are often associated with stress, the high adrenal response does not always reflect a negative condition, as appetitive contexts such as sexual activity and positive arousal can increase cortisol values. Moreover, in both studies, the endocrine response is accompanied by an ethological evaluation that suggests an improved physiological and psychological welfare of great apes (74, 75).

Brent and Eichberg (68) reported the behavioral response of captive male and female chimpanzees (ages 2.5 to 35 years) to a Plexiglas puzzle with holes, where animals could retrieve food rewards (three flavors of PRIMA treats) for over 10 weeks. The board was attached to the roof of the enclosure, and food fell through the holes when chimpanzees used their fingers or tools to retrieve it. The authors found that affiliative (approach, groom, play, or touch) and aggressive (bite, chase, hit, or pant-hoot) behaviors were significantly reduced (to a frequency of 5 and 1%, respectively). Activity and atypical behavior (e.g., arm waving, feces ingestion, feces smear, among others) did not significantly change; however, the animals shifted from sedentary behaviors (e.g., inactivity, self-grooming) to climbing the fence and manipulating the enrichment. Moreover, although animals successfully retrieved all the food treats in 84% of the trials, and the usage of the enrichment did not decrease with time (~17.7% of use each time), frustration responses were also recorded in six chimpanzees, who repeatedly banged on the board to obtain the food rewards (0.58 per subject during the interaction with the puzzleboard). Indeed, Yamanashi et al. (76) report that CE has emotional consequences in laboratory chimpanzees, as indicated by consecutive errors or task difficulty, with incorrect trials leading to an increase in the presentation of self-directed behaviors.

Designing CE that requires tools to obtain food is of particular interest to great apes as it is part of their natural repertoire (77). However, their use varies by individual and species. For example, Masi (78) noted that, compared to bonobos and gorillas, chimpanzees possess the highest manipulation abilities because their primary diet is frugivorous, which requires tool-assisted feeding. In this context, Celli et al. (62) observed female chimpanzees housed at a wildlife park during 10 consecutive days of involving a simulation of an ant-fishing task for 10 consecutive days. The authors provided several tools to retrieve honey from polyethylene bottles (e.g., metal chains, plastic spoons, metal pins, chopsticks, etc.). The bottles were placed in acrylic boxes attached to the front of the animals' cage, so they were out of reach of the chimpanzees, but the bottle openings were accessible. It was found that inactivity decreased by 52%, while foraging increased by 31%. Aggressive behaviors accounted for 1.5% of the activity budget and mainly occurred when juveniles lacked access to the tools. Affiliative behaviors were rarely recorded (1.7%), and EE did not affect the frequency of atypical behaviors, including coprophagy, hair pulling, or stereotyped locomotion (2.5%). In the same species, Morimura (79) implemented acrylic tube feeders to evaluate voluntary tool use in male and female infant laboratory chimpanzees (Figure 2b). When provided with a juice-holding device that could be accessed either by hand or with tools (containing diluted orange juice), the authors found that chimpanzees preferred to use tools to obtain the juice, particularly a straw (98.7%), over using their mouths or hands. This type of CE stimulates species-specific behaviors and provides freedom of choice.

The importance of using tools was also highlighted in western lowland gorillas, who received a stripped piece of bamboo to obtain feed from a boomer ball stuffed with forage for 5 days (66). By providing this enrichment, inactivity scores significantly reduced (from 22.6 to 3.7) while foraging increased (from 4.5 to 19.5) (66). However, Harrison and Whiten (80) reported limited behavioral flexibility when male and female zoo chimpanzees were presented with an artificial foraging task (to obtain juice). The authors provided different tool materials (sticks, straw bedding material, strips of cloth, etc.) and found that chimpanzees selected leafy sticks 50% of the time. This might be related to what Bandini and Harrison (81) mention, that both wild and captive chimpanzees possess an impressive problem-solving ability, but are also highly conservative when creating puzzle solutions.

Although several benefits have been associated with the use of CE, some studies have reported no influence on the presence of atypical behaviors. In this sense, Bloomstrand et al. (82) evaluated the effect of providing male and female chimpanzees housed in a semi-free-ranging park with a food puzzle box filled with peanuts. Each shelf had holes where chimpanzees could insert their fingers to drop the peanuts to the next lower level. The authors reported that after 19 days of enrichment, one individual exhibited a lower frequency of atypical behaviors (from 17 to 6) while another female increased her atypical behavior (frequency from 1 to 6), which may be related to the latter female's low ranking. Similarly, Padrell et al. (36) developed a foraging device for two groups of male and female sanctuary-housed chimpanzees and evaluated it over 3 months. The device consisted of a steel vertical maze filled with food rewards (dried fruits and nuts). Chimpanzees obtained food through a series of holes at the front of the device, where they could insert sticks or branches. Although inactivity time decreased, atypical behaviors (rocking, pacing, self-harm, coprophagy, regurgitation, re-ingestion) did not decrease when compared to baseline (fewer than 1%). Moreover, the interaction time with the enrichment device decreased over time, which might be related to frustration by not obtaining food rewards after failed attempts.

The same authors also reported the lack of influence of the EE on stereotypical behaviors in a subsequent study conducted with sanctuary-housed chimpanzees. Padrell et al. (83) implemented an artificial termite-fishing task to enrich the enclosure of male and female chimpanzees aged 15 to 35 years. PVC tubes simulated the mound holes and were filled with honey, peanut butter, and muesli. The authors collected behavioral data and found that both males and females participated in enrichment tool use, and feeding increased (by a proportion of up to 0.8 scans). In comparison, inactivity decreased (by 0.1). However, stress-related behaviors (e.g., rocking, pacing, scratching, rubbing) did not change (significantly initial frequency: 2.5%). As a result of past traumatic experiences, great apes can have fixed stereotypies that are difficult to eradicate even when providing adequate environments (36). Therefore, the individual history of animals must also be considered (individual differences are also discussed below).

In recent years, technological advancements have been merged with CE for great apes by incorporating interfaces and other digital alternatives to create a highly sensorial enclosure (72, 73). These alternatives include those where animals participate passively (e.g., watching videos) or actively (e.g., touchscreen tablets, motion sensors, and virtual interactive activities) (84). For example, Bloomsmith and Lambeth (85) studied the effects of videotapes of humans, chimpanzees, a black screen, and other animals on the behavioral responses of male and female chimpanzees housed in a science park. The videos were projected in color and sound on 19-inch monitors. Although behavior did not change when exposed to audiovisual stimuli, individually housed animals watched the monitor significantly more (67.3 vs. 19.4%) than group-housed chimpanzees. Moreover, the animals habituated to the monitor over time. In the same species, Boostrom (86) researched the use of an iPad™ for 6 months in both zoo orangutans and chimpanzees (male and female). Animals interacted with the tablet for 5 min twice per month. The tablet included applications to promote sensory and tactile enrichment, such as GT Zoo, Music Sparkles, Painting Sparkles, Koi Pond, Farm Sounds, Cat Fishing, and other games for cats. Chimpanzees were presented with multiple applications during each experimental session. It was shown that animals preferred applications with auditory stimulation (up to 5 min of interaction time). Additionally, juveniles showed the most interest in the iPad, but both species preferred brightly colored applications that also provided auditory stimulation (e.g., music and painting sparkles).

**Figure 5:**
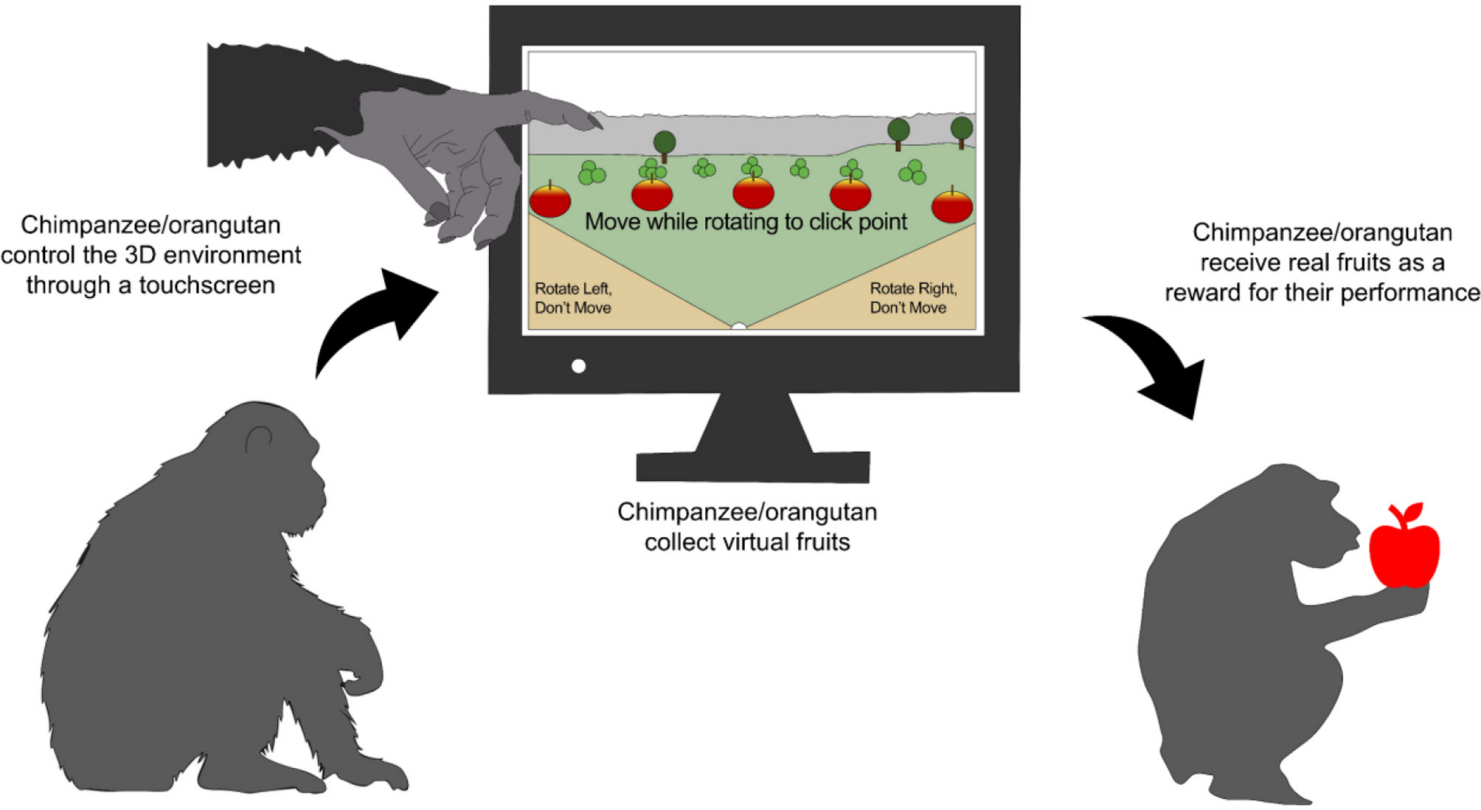
Virtual 3D environments as cognitive enrichment for great apes. great apes are species that can interact with several technologies, such as touchscreen devices. 3D environments stimulate the animal's problem-solving and association skills to obtain the reward. Original image made with information retrieved from McEwen et al. (28).

Handheld tablet devices and 3D virtual environments (called APExplorer_3D) have also been explored with chimpanzees and orangutans by McEwen et al. (28). Animals, with and without touchscreen experience, were trained to interact with a 100 m^2^ virtual environment that encouraged foraging through the projection of virtual fruits. Apes moved an agent through the environment (a grassy area with a lake and trees) to collect fruit by touching the screen. When animals collected the fruit in the screen, real fruit rewards were provided (Figure 5) (28). The findings demonstrated their ability to interact positively with these complex environments and learn spatial connections, as interactions with the monitor progressively increased, exceeding the record of 100 positive responses (contact with the monitor) per day from the 50th session onwards. Regarding virtual technologies, Yamanashi et al. (87) used video technologies to expose zoo chimpanzees to a virtual forest presented as an interactive movie with motion-tracking sensors. Behavioral evaluations showed that chimpanzees positively responded to movie scenes by playfully interacting with them, particularly young chimpanzees. Likewise, Grunauer and Walguarnery (88) compared the effects of tactile enrichment (painting on paper) and virtual painting with using an iPad™ (called Kaleidoscope drawing pad) on male and female chimpanzees in a zoo. Both enrichments decreased the basal frequency of self-picking (5.12/10 min) when painting (1.42/10 min) and when using the iPad™ (1.86/10 min). Moreover, displacement behaviors such as yawning decreased from 5.21/10 to 0.17/10 min with both enrichments.

The effect that CE has on great apes is also highly influenced by gender. In the first instance, as stated by Padrell (36), female chimpanzees seem to engage with CE more frequently than males (between 15 and 50% of the scans). Similarly, when comparing the use of a puzzleboard in male and female chimpanzees, females used the puzzle more often than males (up to 70%) (68). Secondly, gender is related to the ability of animals to understand the task, with females mastering new tools faster than males (68). Individual differences might also be recorded, and animals might have preferences, as mentioned by Pruetz and Bloomsmith (89), who found that captive chimpanzees prefer destructible wrapping paper over Kong Toys. Moreover, Gilloux et al. (90) found differences between species, with zoo chimpanzees using the puzzle feeder more (18%) than gorillas and orangutans (10 and 9.4%, respectively).

The different responses recorded in great apes can also be related to experience or prior knowledge, as shown in the sanctuary and zoo male and female chimpanzees (91). The study found that animals with prior experience using the enrichment device (including food items inside small cardboard tubes hidden within a larger tube) had longer use times (up to 48.9 min/h) that were first exposed to the enrichment (as low as 7 min/h). However, the total time of use increased after the first success. Therefore, the duration of exposure to the enrichment needs to be considered to observe beneficial effects on the species while avoiding habituation to the device (62, 73). Likewise, complexity and controllability influence the effect that EE has on great apes (61). As their wild counterparts, great apes under human care are highly motivated by food-related enrichments that require problem-solving. These enrichments need to provide certain control over their environment while keeping them engaged in solving puzzles for rewards and cognitively active to promote their mental welfare (36, 46, 79, 92). Table 3 summarizes the main outcomes of implementing CE in great apes.

**Table 3:** Summary of discussed studies addressing CE for great apes.

**Species**	**Setting**	**Device**	**Sample size**	**Duration**	**Outcomes**	**References**
Bonobos (*Pan paniscus*) Western lowland gorillas (*Gorilla g. gorilla*) Sumatran orangutans (*Pongo abelii*)	Zoo	Boxes, hoses, or tennis balls baited with treats	12 9 7	2 years	↑ Salivary cortisol	Behringer et al. (74)
Chimpanzees (*Pan troglodytes*)	Science park	Food puzzles	41	6 months	↓ Agonistic behavior ↑ Feeding ↑ Social grooming	Bloomsmith et al. (65)
Chimpanzees (*Pan troglodytes*)	Science park	Videotapes	10	400 tests	Individually housed animals interacted more than group-housed animals Habituation to the monitor	Bloomsmith and Lambeth (85)
Chimpanzees (*Pan troglodytes*)	Science Park	Food puzzle box	20	19 days	– Atypical behavior	Bloomstrand et al. (82)
Orangutans Chimpanzees	Zoo	iPad™	10 8	6 months	Animals prefer apps with auditory stimulation	Boostrom (86)
Chimpanzees (*Pan troglodytes*)	Research center	Puzzle board	29	10 weeks	↓ Affiliative behavior ↓ Aggression – Atypical behavior	Brent and Eichberg (68)
Chimpanzees (*Pan troglodytes*)	Sanctuary Zoo	Puzzle feeder	11	N/S	↑ Use time	Brooks et al. (91)
Chimpanzees (*Pan troglodytes*)	Primate park	Ant-fishing task simulation	6	10 days	↓ Inactivity time ↑ Foraging – Atypical behavior	Celli et al. (62)
Western lowland gorilla (*Gorilla g. gorilla*)	Zoo	Cuboid puzzle maze	6	6 months	↑ Tool use No aggression	Clark et al. (73)
Western lowland gorilla (*Gorilla g. gorilla*)	Zoo	Cuboid puzzle maze	7	120 min	Interest in enrichment ↑ Finger use	Gray et al. (72)
Chimpanzees (*Pan troglodytes*)	Zoo	Painting on paper Virtual painting	8	2 months	↓ Self-picking ↓ Displacement behavior	Grunauer and Walguarnery (88)
Chimpanzees (*Pan troglodytes*) Orangutans (*Pongo abelii*)	Zoo	3D virtual environment	5 6	N/S	Learned spatial connections	McEwen et al. (28)
Chimpanzees (*Pan troglodytes*)	Research institute	Acrylic tube feeders	4	30 sessions	↑ Tool use	Morimura (79)
Chimpanzees (*Pan troglodytes*)	Sanctuary	Foraging device	14	3 months	↓ Inactivity time – Atypical behavior	Padrell et al. (36)
Chimpanzees (*Pan troglodytes*)	Sanctuary	Artificial termite-fishing tank	14	640 scans	↑ Feeding ↓ Inactivity time – Stress-related behavior	Padrell et al. (83)
Western lowland gorilla (*Gorilla g. gorilla*)	Zoological park	Boomer ball stuffed with forage	6	5 days	↓ Inactivity time ↑ Foraging	Ryan et al. (66)
Chimpanzees (*Pan troglodytes*)	Zoo	Mechanical mazes	10	10 trials	Problem-solving skills	Vlaming et al. (64)
Bonobos (*Pan paniscus*) Orangutans (*Pongo pygmaeus*) Gorillas (*Gorilla gorilla*)	Sanctuary	Computerized mazes	4 7 6			
Chimpanzees (*Pan troglodytes*)	Zoo	Artificial termite mounds	9	6 weeks	↓ Inactivity time	Zaragoza et al. (59)
Gorillas (*Gorilla g. gorilla*)		Nautical hollow buoys	4		↓ Atypical behavior ↑ Feeding ↑ Social behavior ↑ Exploratory behavior ↑ Salivary cortisol	
Chimpanzees (*Pan troglodytes*)	Zoo	PVC pipe feeder	5	5 months	↓ Self-directed behavior ↓ Inactive time	Yamanashi et al. (69)
Chimpanzees (*Pan troglodytes*)	Zoo	Virtual forest	6	14 days	Playful interaction with the virtual environment	Yamanashi et al. (87)

↑, significant increase; ↓, significant decrease; –, no change; N/S, not specified.

## 6 Frustration and aggression when providing enrichment to non-human primates

In general, providing NHPs with adequate CE is associated with several behavioral and physiological benefits. However, some studies have addressed the negative responses that enrichment devices may elicit in NHPs (e.g., frustration and aggression). Mason et al. (93) mention that frustration-induced stereotypic behaviors are elicited by motivational frustration. While NHPs are naturally exposed to challenging environments, when they face challenges with limited resources or a lack of skills to solve them effectively, frustration might arise (94). This is similar to when animals are exposed to complex, insolvable, or inescapable tasks leading to frustration, distress, and other negative outcomes.

For example, Tecwyn et al. (14) evaluated the performance of zoo-housed bonobos and orangutans when interacting with a paddle box of varying difficulty levels. The box, attached to the outside of the enclosure, required animals to rotate the paddles, with wooden handles, to obtain a food reward (orange, apple, pear, or bread). The difficulty of the interaction was that in some instances, the animals could retrieve the reward by rotating one or two paddles, or by doing so in a single movement. The authors reported that 3- or 2-step trials were associated with poor performance, while a 1-step trial had a performance of 97.9% of correct trials. Moreover, it was observed that orangutans and bonobos were significantly less likely to retrieve food when it was placed on the top level of the paddlebox, possibly due to higher cognitive demand and lower motivation to obtain the reward.

Frustration responses were reported by Lyons et al. (95) in squirrel monkeys (*Saimiri sciureus*) when the location of their food was changed. While cortisol increased immediately after the change, monkeys that were able to locate the new foraging sites significantly reduced their cortisol levels (~130 μg/dl). In contrast, monkeys that were unable to locate the food showed a significant increase in cortisol levels (~200 μg/dl). However, increases in cortisol are not always associated with distress and might reflect enhanced cognition. This was reported by Woo et al. (96), who found that showing pictures of monkeys, humans, or animations increased the cortisol levels in cynomolgus monkeys (from 30 to up to 40 μg/dl), but this was correlated with the animals' cognitive function.

Aggression is another outcome that might be observed when providing enrichments to NHPs. For example, some studies in male and female orangutans (*P. pygmaeus*) have reported that computer-joystick enrichment resulted in aggressive and anxiety-related behaviors (97). These responses might be lessened by providing multiple devices to avoid competition, as Mallavarapu et al. (98) recorded in zoo orangutans. These authors evaluated the response of male-female pairs of orangutans to multiple automatic reward dispensers. The device consisted of a monitor computer connected to a joystick and an automatic reward dispenser. Animals were rewarded with amounts of General Mills Kix^®^ cereal when completing four tasks successfully: side, chase, easy maze, and complex maze tasks. The results showed no aggression, rough scratching, or atypical behaviors when multiple joystick systems were provided during the enrichment period.

Increased contact and non-contact aggression was also reported in zoo-housed Japanese macaques when interacting with two touchscreen devices, probably due to the monopolization of the devices (56). An alternative to reduce aggression and trauma was proposed by Wooddell et al. (99), who reported that produce enrichment (corn-in-husk, whole melon, or squash) reduced the risk of trauma in laboratory rhesus macaques. The total trauma was reduced by 38%, and hospitalization was reduced by 47%. This relates to the time required to process and extract food, engage animals in foraging activities, and reduce socially derived injuries. However, this enrichment did not reduce the presence of severe trauma, which might be related to the competition for food.

Negative responses (e.g., frustration and aggression) can be elicited by an enrichment program. To prevent these adverse effects, when designing CE for great apes, factors such as the hierarchy, species, sex, age, and the animal's individual background must be considered (100). Poorly distributed enrichment is often associated with increased aggression because food-related rewards become a defendable resource (66), Providing animals with multiple and similar enrichments might decrease defense aggression by allowing different individuals to interact with the enrichment device simultaneously (50). Table 4 presents the main issues that may arise during CE implementation, along with recommendations to address them.

**Table 4:** Suggested aspects that must be considered as design guidelines on how to implement CE in NHPs.

**Species**	**Issue**	**Recommendation**	**References**
Japanese macaques	Aggression	Provide multiple devices	Jacobson et al. (56)
Squirrel monkeys	Frustration	Evaluate the physiological response of animals	Lyons et al. (95)
Orangutan	Aggression	Provide multiple devices	Mallavarapu et al. (98)
Orangutan	Aggression	Device duplication	Tarou et al. (97)
Bonobos Orangutans	Enrichment difficulty	Adjustably difficulty	Tecwyn et al. (14)
Rhesus macaques	Aggression	Produce enrichment	Wooddell et al. (99)

## 7 Applications and challenges in captive settings

### 7.1 Implementation in research laboratories

Following the Animal Welfare Act (AWA) in 1985 and the publication of “The psychological wellbeing for nonhuman primates” in 1998, EE programs in laboratory primates have gained importance due to their highly cognitive abilities (101). The AWA mentions that research facilities need to “provide a physical environment adequate to promote the psychological wellbeing of primates” (92). According to the National Research Council (NRC), standard primate husbandry protocols and refined care for laboratory animals must provide complex environments that minimize harm or distress and maximize animal welfare from birth to death (102–104).

As Lutz and Novak (46) mention, NHPs are social species that require interaction with conspecifics to meet their behavioral needs. The Guide for the Care and Use of Laboratory Animals states, “With respect to NHPs, the Committee endorses social housing as the default…” (105). Additionally, AAALAC International states “Primates are social species; thus, must be socially housed and enclosures must permit pair or group housing of animal” and takes the position “Social housing will be considered by AAALAC International as the default method of housing unless otherwise justified based on social incompatibility resulting from inappropriate behavior, veterinary concerns regarding animal wellbeing, or scientific necessity approved by the IACUC (or comparable oversight body)” (106). Although most research primates are socially housed in federally funded and/or AAALAC-accredited institutions, some instances require individually housing animals (107–111). This hurts the animal's welfare and makes EE implementation challenging (4). Even when individually housed, captivity must stimulate species-typical behavior (112). In this sense, CE is considered a key component in research facilities, as highlighted in Richardson's (113) review, which found that, from 52 papers focused on CE for different types of NHPs (monkeys and great apes; covering from 1978 to 2019), 71% of the research was performed in laboratory settings.

The AWA emphasizes the need to provide social, physical, or sensory enrichment. However, when these cannot be provided or the implementation of puzzles is a constraint on the research, positive reinforcement training (PRT) has been considered a type of CE as NHPs learn while interacting with humans (112, 114). For example, Perlman et al. (115) highlight that PRT improves voluntary animal cooperation in specific procedures such as blood collection or vaccines. It also provides opportunities for choice and control, inherent aspects of CE for NHPs. Additionally, new automated systems for PRT as CE have been developed by Tulip et al. (116) in housed macaque monkeys. The authors obtained high performance levels (~80%) when training female rhesus macaques to press buttons of varying difficulty to obtain a reward. Nonetheless, surveys have reported that nearly 50% of researchers believe PRT might interfere with the research, while 63% report that the cost is a concern when deciding to implement animal training programs (115).

In other instances, CE is part of the research protocol, as in neuroscience research. An example is Bliss-Moreau and Baxter's (117) study, in which rhesus monkeys manipulated a food puzzle to evaluate cognitive capacity by age. Likewise, studies focusing on the functional characterization of cortical areas in macaque monkeys have used bimanual precision grip and reach tasks to obtain a food reward (118). Food puzzles using marshmallows as rewards were also used to examine the correlation between the rhesus monkeys' problem-solving and two rearing conditions, either canine companions or inanimate substitute mothers (119). In these studies, although puzzles were used to evaluate cognitive tasks for research purposes, they can serve as CE for NHPs.

When designing an EE protocol for research animals, the species, age, sex, personnel time, cost, and individual history must be considered along with the experimental protocol (120). Considering the experimental protocol is essential, as most cases require compliance with specific conditions (e.g., individual housing). Moreover, as mentioned by Coleman and Novak (92), the desired outcome is a critical aspect of developing an appropriate EE protocol (e.g., behavioral, physiological, or cognitive outcomes). Designing an appropriate CE protocol not only provides benefits to the animal but also confers validity and increases the control of the experimental setting. By meeting NHPs' behavioral needs and their advanced cognitive demands, researchers can obtain robust data while encouraging welfare (46, 101, 121).

### 7.2 Application in zoos and conservation centers

Research on cognitive abilities in animals housed in zoos and conservation centers has attracted increasing interest in recent decades due to its impact on improving animal welfare through CE. As mentioned by Hopper et al. (9), this research enables both the understanding of complex cognitive abilities and the design of more dynamic environments that promote natural behaviors and provide significant benefits during the management and conservation of NHPs under human care in zoos. Although zoo enclosures are specially designed for the species, captive animals reside in environments that partially challenge their cognitive abilities (37). Therefore, a practical strategy is the application of tools that meet their cognitive needs. In this sense, zoos provide the opportunity for cognitive research due to the continuous access to wild species under human care and the possibility of implementing controlled experimental protocols with low stress for the animals (9).

In the case of prosimians, Stoinski et al. (26) evaluated social learning in black-and-white collared lemurs (*Varecia variegata*) using a stimulus-reward association learning device applied during feeding. The tube was filled with food, and the primate was required to lift or slide a lid to obtain the reward. The results showed the adoption of the previously observed technique, when lemurs manipulated the apparatus more than 30% of the time after training, demonstrating social learning in a species where this ability had been underestimated. Moreover, in other species of monkeys (crested macaques), Micheletta et al. (122) observed that face recognition is influenced by social variables such as hierarchy, as subjects performed better when the images corresponded to dominant individuals (dominant), resulting in 20% more successful responses than with subordinate individuals (subordinate).

In monkeys, Day et al. (15) conducted a comparative study on neophilia, innovation, and social attention in callitrichid species, including the lion tamarin (*Leontopithecus*), tamarins (*Saguinus*), and marmosets (*Callithrix*). Using novel extractive foraging tasks, they observed that lion tamarins consistently exhibited shorter response latencies (10 vs. 20 min) and higher levels of successful handling (frequency 2) compared to cotton-top tamarins (0.2) and marmosets (1), suggesting that species with diets that require more handling are less neophobic and more innovative. It is especially worth noting that in small, arboreal, and neotropical species, the extractive foraging style is a morphological and behavioral specialization that responds to entomophagy. This involves a diet based on insects and small vertebrates, requiring a search for prey hidden on hard-to-reach surfaces using their slender hands and fingers.

In great apes, Talbot et al. (123) investigated face discrimination skills in orangutans using images of familiar conspecifics and unfamiliar heterospecifics through a simultaneous matching-to-sample paradigm and socio-cognitive processes tasks. The results indicated that orangutans were significantly better at discriminating conspecifics (80%) than heterospecifics (60%). Furthermore, Scheumann and Call (27) assessed spatial memory skills in Sumatran orangutans (*P. abelii*) and yellow-cheeked gibbons (*Nomascus gabriellae*). Researchers placed portions of kiwi, banana, and grapes at 10 locations within a 1,900 m^2^ area. In each session, the number of locations visited or recalled by subjects was recorded compared to the baseline session. Individuals of both species remembered the location of previously discovered food items, with the percentage of visited locations progressively increasing from 0 to 60% starting from the 13th session. Some orangutans even associate locations with specific types of food (foraging decisions), showing a preference for kiwifruit. This is crucial for arboreal species with dispersed diets that devote a considerable percentage of their time and energy to foraging in natural habitats.

Other highly relevant findings in social species regarding problem solving in hierarchical structures (problem solving in the physical domain) are demonstrated in the study of bonobos and orangutans (*P. pygmaeus*), which revealed that great apes can solve tasks step by step. Using rotating paddles to retrieve a reward from a target location, the results suggest that they are capable of sequential reasoning, demonstrating up to 80% success in solving the cognitive challenge, and indicating they have the cognitive capacity to anticipate the consequence of their motor actions (14).

**Figure 6:**
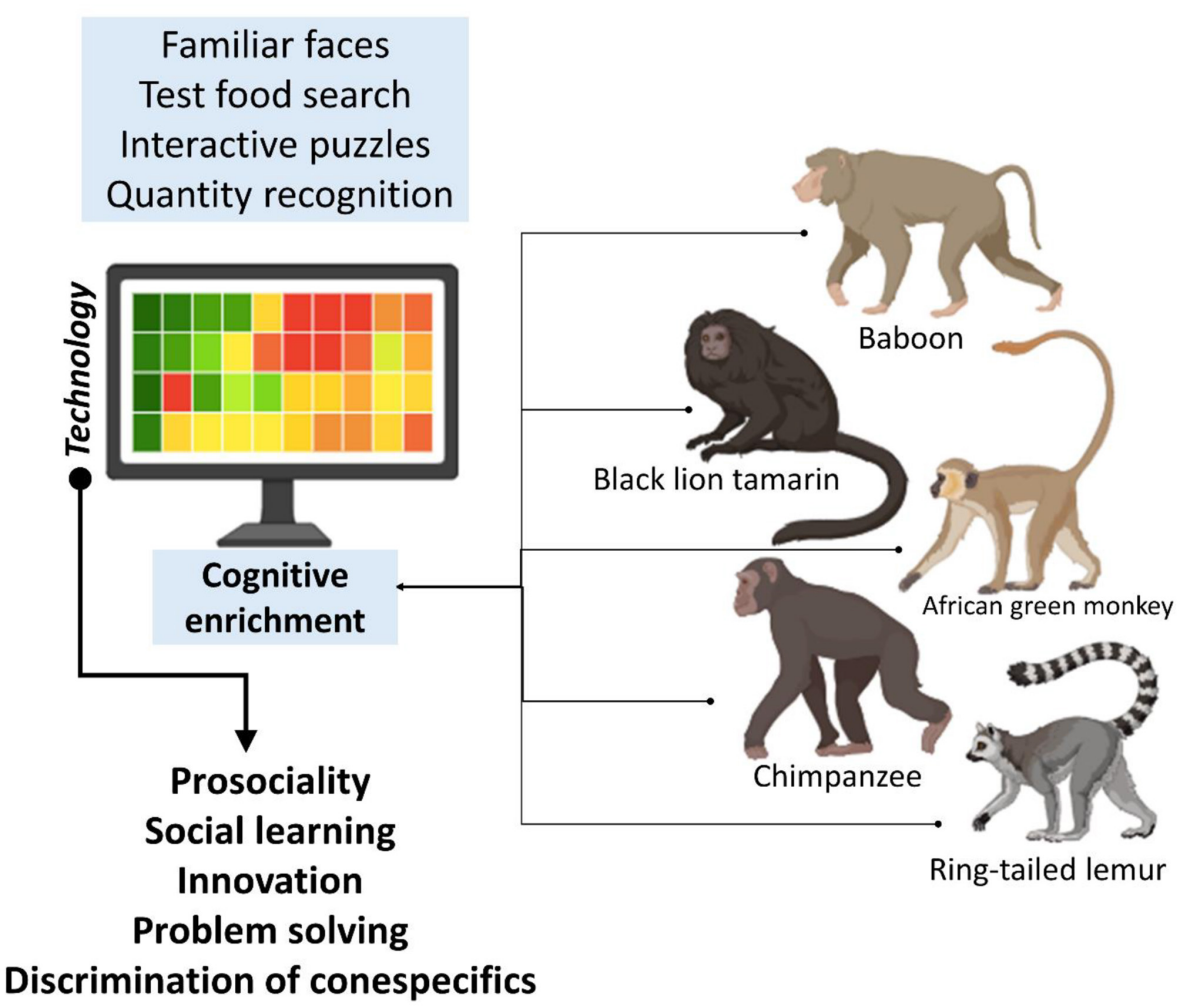
Use of technology and cognitive enrichment in non-human primates.

On the other hand, in the field of technological tools for cognitive study, Perdue et al. (124) developed the computerized evaluation system “Rumbaughx” to study metacognition, numerical and quantitative cognition, and inhibitory brain mechanisms (game theory) in different primate species (Figure 6). This automated system collects data and reduces the presence of researchers, optimizing animal welfare and the objectivity of studies by the controlled and stimulating evaluation environment (125). Applied science through technology can evaluate cognitive ability in non-human primates and has revealed the level of quantitative discrimination in species such as gorillas which are subjected to a system for approximate number estimation. This demonstrates their capacity for learning through training (“joystick trained”).

Furthermore, it has been documented that social environments and the presence of visitors can influence the performance of CE strategies. Huskisson et al. (7) evaluated the effect of a human audience on Japanese macaques with touchscreen-enriched environments using a zero-delay identity match-to-sample task. They found that, although their interaction with touchscreens did not decrease with the presence of visitors, their response latencies varied significantly (2.77 closed vs. 2.80 open, long transformed mean). When the number of visitors did not exceed 20, 67.45% of positive interactions with the device were recorded, compared to those recorded during a visit with a group of more than 20 visitors (22.15%). This suggests a subtle moderation of attention and emotional state during cognitive testing in zoo animals.

These studies suggest that CE not only promotes the emotional and behavioral welfare of primates but can also promote the development and expression of complex cognitive abilities such as social learning, tool use (task behavior), and decision-making (planning and explorative behavior) based on the observation of others (allomimicry) (7–9). These findings contribute to replicating ecological and social conditions closer to the natural environment, which is especially valuable for species undergoing reintroduction or *ex-situ* conservation programs. However, the study of cognitive stimulation in NHPs faces complexities, such as the need to design protocols that include less-studied species and adapt activities and devices to the anatomical, physiological, cognitive, and even behavioral characteristics of each species that prevent negative states such as frustration or agonistic encounters (9, 29). Likewise, a more systematic assessment of the long-term impacts of CE is required, both on behavior and on physiological indicators of wellbeing (10, 29).

Implementing CE in zoos and conservation centers represents a fundamental link between animal welfare and scientific research. This approach not only enriches the lives of animals under human care but also provides crucial information on the evolution of cognition and culture in NHPs. A focus on diversifying the studied species, using noninvasive cognitive monitoring technologies, and implementing enrichment programs integrated into long-term management and conservation plans is suggested.

### 7.3 Use in sanctuaries and rescue facilities

Currently, thousands of great apes live in captivity, distributed mainly in zoos, research facilities, and sanctuaries. It is estimated that ~3,500 individuals are registered in zoos and sanctuaries worldwide (126). In Africa, 23 sanctuaries are registered, housing ~3,000 NHPs (127). The welfare of these animals varies considerably within and between different types of captivity, with no clear and consistent correlation between housing type and welfare level (37). Sanctuaries and rescue centers focus primarily on the welfare of NHPs, providing a permanent home to rescue animals from abuse, illegal trafficking, the pet industry, illicit research laboratories, or entertainment (128). Most animals in sanctuaries have complex life histories, often involving physical or psychological trauma, which directly influences their biological or management needs (129). Therefore, the implementation of CE strategies represents a valuable alternative for addressing and reversing behavioral abnormalities in individuals with these backgrounds (37).

Some studies have explored the application of CE in chimpanzees (Pan troglodytes) housed in sanctuaries. Padrell et al. (83) investigated the impact of artificial termite-fishing tasks, a tools-tasks based enrichment activity. These findings take on great relevance by consolidating the scientific evidence surrounding the optimization of cognitive skills and the recovery from possible brain damage or dysfunction resulting from environmental enrichment; since a restoration of synaptic connectivity and neurogenesis caused by possible pathological events is encouraged (22).

A recent study examined the short- and long-term consistency of handedness preferences and hemispheric laterality in sanctuary chimpanzees using unimanual and bimanual coordinated tasks. Although not considered enrichment, the tube occupational task represented a cognitive and motor challenge. The results showed that the direction of handedness in bimanual activities remained stable throughout the observation period, with a clear preference, which, it is suggested, could be a consequence of practice and experience gained during use of the device. This type of research is essential for understanding how cognitive and motor skills can be influenced, preserved, and improved in captive nonhuman primates, considering individual needs determined by anatomy, physiology, and cerebral hemisphere dominance (130).

In this context, the application of CE to non-human primates in sanctuaries and rescue centers is crucial to promote their wellbeing and the expression of natural behaviors, while facilitating research on their cognitive abilities (9). Implementing tool-based tasks can significantly increase the time allocated to natural and species-specific behaviors, thereby fine-tuning the use of species-specific skills (5, 37).

## 8 Conclusions

Non-human primates are species that possess significant cognitive abilities, including complex learning, memory, problem-solving, and tool use. Their sophisticated cerebral processing requires a stimulating and challenging environment that replicates what NHPs encounter in the wild. Thus, NHPs housed in zoos, conservation centers, sanctuaries, or research facilities require a cognitively stimulating environment to preserve their welfare and avoid the negative consequences of captivity (e.g., atypical behaviors and stereotypies). In this sense, CE, a type of EE in which the cognitive functioning of animals is stimulated through objects, tasks, or puzzles, has been shown to provide benefits to NHPs by reducing the activation of stress-related axes and decreasing the presentation of behavioral responses such as self-injuries, repetitive behaviors, regurgitation, and hair plucking, among others. To date, CE has been applied to several primate species. However, to enhance its impact as an enrichment strategy, we propose expanding the range of species analyzed. Although all are non-human primates, wide variability between species has been demonstrated. In addition, incorporating non-invasive technologies for cognitive monitoring and integrating these programs into long-term management and conservation policies is essential. When designing CEs for NHPs, factors such as the device difficulty and the number of devices must be considered. Animals should be presented with cognitive challenges of different difficulty so they can decide whether to participate, preventing frustration or boredom. Moreover, in socially housed species, defense aggression can be prevented by placing several devices or items so that all individuals can interact with the enrichment device simultaneously.
